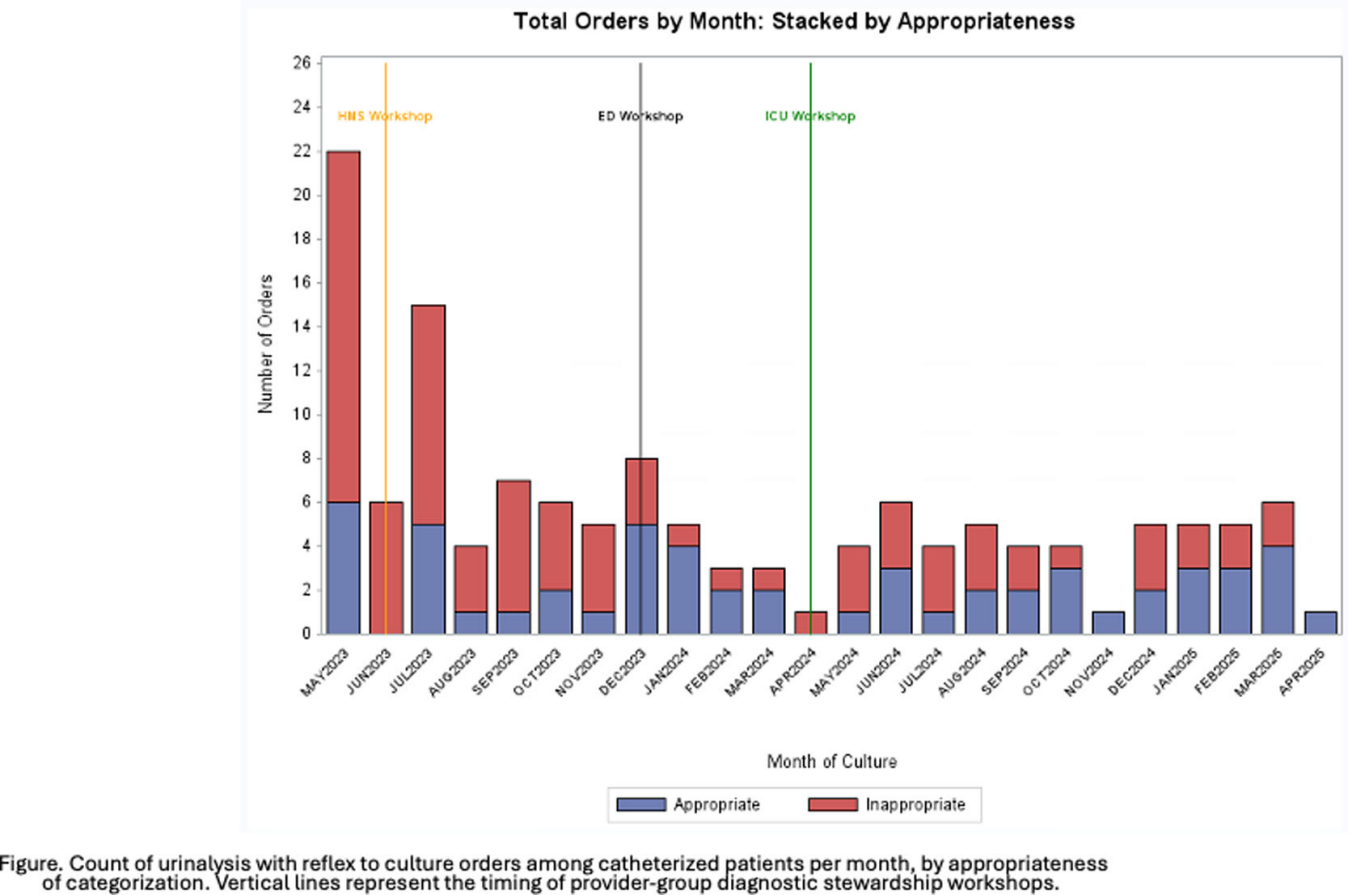# 200 Cardiac and Endovascular Implant Infections: Impact of Timing of ID consultation on Hospital Length of Stay?

**DOI:** 10.1017/ash.2026.10588

**Published:** 2026-06-23

**Authors:** Gabrielle Schaller, Marissa Morales, Krystle Johnson, Sarah Moore, Hasan Shabbir, Patty Rider, Nourhan Ismaeel, Scott Fridkin, Lucy Witt

**Affiliations:** 1 Emory University- Rollins School of Public Health; 2 Emory Healthcare; 3 Emory University

## Abstract

**Background:** Inappropriate urine culture ordering among catheterized patients can result in overdiagnosis of catheter-associated urinary tract infections (CAUTI) and unnecessary antibiotic exposure. We sought to reduce inappropriate urine culture ordering at our 167 bed acute care hospital with targeted diagnostic stewardship educational initiatives to hospitalist, emergency room, and intensive care unit providers (ICU). Objective: Estimate the impact of educational workshops on appropriate urinary ordering practices among inpatient providers at a single small acute care hospital. **Methods:** Retrospective observational cohort study of all catheterized patients receiving a urinalysis with reflex to culture from March 2023-April 2025. Workshops were held during different months tailored to provider groups, and attendance was recorded. Categorization of order indications and appropriateness were assessed retrospectively by a study team member applying established algorithms (i.e., IDSA guidelines with minor modifications). Patient clinical and encounter data were extracted from the electronic health record. Indications and appropriateness of orders were compared between provider groups and by provider attendance at a workshop by univariate and multivariate analysis. **Results:** Of 139 urinalyses with reflex to culture orders, 63 (45%) were ordered by Hospitalists, 40 (29%) by critical care providers, and 36 (26%) by other provider groups; 81 (59.6%) were inappropriate, without differences in appropriateness by provider group (p=0.10). Frequency of ordering decreased over time (7.2/month prior to last workshop vs. 4.2/month after, Figure); however, the proportion categorized as inappropriate appeared stable over time (Figure). Appropriate orders were mostly for UTI symptoms without fever (14, 25.5%), with fever (10,18.2%), or sepsis (16, 29.1%). Provider workshop attendance was as frequent among appropriate orders as inappropriate (25/55, 46% vs. 26/81, 32%, p=0.12). There were no significant differences in catheter duration, comorbidity score, and various primary discharge diagnosis by order appropriateness. **Conclusion:** Over two years, a majority of urinalysis with reflex to culture orders among catheterized patients were inappropriate. While diagnostic stewardship workshop attendance did not appear to impact likelihood of an order to be appropriate, we did observe an overall decrease in orders suggesting educational efforts may have influenced ordering behavior.

**Figure:**